# Synthetic lethal interaction between the tumour suppressor *STAG2* and its paralog *STAG1*

**DOI:** 10.18632/oncotarget.16838

**Published:** 2017-04-05

**Authors:** Lorena Benedetti, Matteo Cereda, LeeAnn Monteverde, Nikita Desai, Francesca D. Ciccarelli

**Affiliations:** ^1^ Division of Cancer Studies, King's College London, London SE1 1UL, UK; ^2^ Cancer Systems Biology Laboratory, The Francis Crick Institute, London NW1 1AT, UK

**Keywords:** synthetic lethality, cohesin complex, paralog dependency, cancer vulnerability, precision medicine

## Abstract

Cohesin is a multi-protein complex that tethers sister chromatids during mitosis and mediates DNA repair, genome compartmentalisation and regulation of gene expression. Cohesin subunits frequently acquire cancer loss-of-function alterations and act as tumour suppressors in several tumour types. This has led to increased interest in cohesin as potential target in anti-cancer therapy. Here we show that the loss-of-function of *STAG2*, a core component of cohesin and an emerging tumour suppressor, leads to synthetic dependency of mutated cancer cells on its paralog *STAG1*. *STAG1* and *STAG2* share high sequence identity, encode mutually exclusive cohesin subunits and retain partially overlapping functions. We inhibited *STAG1* and *STAG2* in several cancer cell lines where the two genes have variable mutation and copy number status. In all cases, we observed that the simultaneous blocking of *STAG1* and *STAG2* significantly reduces cell proliferation. We further confirmed the synthetic lethal interaction developing a vector-free CRISPR system to induce *STAG1*/*STAG2* double gene knockout. We provide strong evidence that STAG1 is a promising therapeutic target in cancers with inactivating alterations of STAG2.

## INTRODUCTION

Cohesin is an evolutionarily conserved complex composed of four core proteins (SMC1A, SMC3, RAD21 and either STAG2 or STAG1) that form a ring-shaped structure able to encircle chromatin [1]. In somatic cells, cohesin is responsible for the cohesion of sister chromatids and proper chromosome segregation during mitosis [1, 2]. Besides this canonical role, cohesin is involved in a plethora of other functions including DNA replication and repair, regulation of gene expression and genome compartmentalisation [3, 4]. All cohesin components, except *STAG1*, have tumour suppressor roles in several cancer types, including leukaemia, sarcoma, glioblastoma and bladder cancer [5]. The mechanism by which altered cohesin contributes to cancer is still unclear. It has been proposed that defects in chromatid cohesion may be responsible for cancer aneuploidy and increased genomic instability [3, 6]. However, several cancers with inactivating alterations in the cohesin complex maintain a nearly normal karyotype. This led to speculation that the alteration of other cohesin functions, such as transcriptional deregulation or defective DNA repair, may contribute to cancer [7, 8].

Given their widespread tumour suppressor activity, cohesin proteins are of obvious clinical interest for the development of targeted cancer therapy. However, tumour suppressors are difficult to target directly because they require agonists or activators that are able to rescue the lost functions. An alternative strategy is to target tumour suppressors indirectly by interfering with their synthetic lethal partners. Synthetic lethality indicates a genetic interaction where the concomitant alteration of two nonessential genes leads to cell death while the alteration of either gene individually is viable. Blocking synthetic lethal partners of tumour suppressor genes is a powerful way to selectively kill cancer cells where they are inactive, while the normal cells remain viable because the genes are wild-type (WT) [9]. Synthetic lethality is the outcome of different types of genetic interactions that make the cell resilient to single gene loss, including back up pathways, rewired intracellular networks or functional compensation due to genetic redundancy. Genes that originate via duplication (paralogs) are interesting candidates for functional compensation because paralogs often preserve some degree of redundancy. Therefore, the identification of ‘paralog dependencies’ is an emerging strategy to uncover cancer vulnerabilities of potential relevance in cancer therapy [10].

Here we investigate whether paralog dependency is established in cancers that acquire loss-of-function (LoF) alterations in the cohesin complex. We focus specifically on *STAG1* and *STAG2* because they are duplicated cohesin subunits that are both expressed in somatic cells. Using a variety of experimental approaches and cell lines, we show that *STAG1* and *STAG2* are synthetic lethal partners and provide evidence that *STAG1* is a potential therapeutic target in tumours where *STAG2* is inactive.

## RESULTS

### Evidence of functional compensation between *STAG1* and *STAG2*

**Figure 1:**
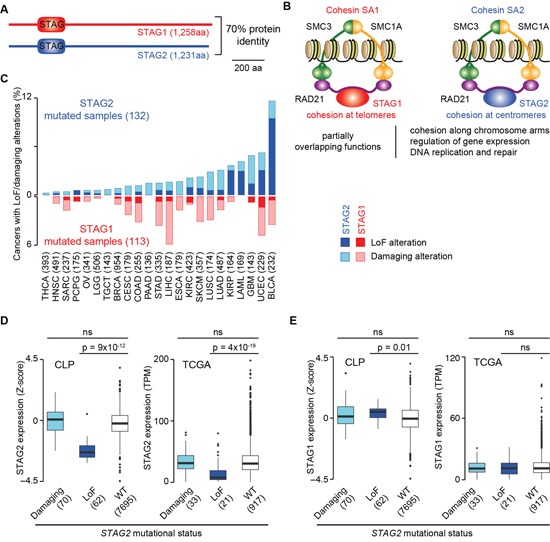
Evidence of functional compensation between STAG1 and STAG2 **Legend: (A)** Sequence identity and domain architecture of STAG1 and STAG2 proteins as annotated in the SMART database [57]. **(B)** Composition and biological functions of cohesin SA1 and cohesin SA2. **(C)** Fraction of TCGA cancers with LoF alterations (homozygous gene deletions, truncating mutations and multiple hits) or damaging missense and splicing mutations in *STAG1* or *STAG2* divided by tumour type. The total number of sequenced samples in TCGA is reported in brackets. BLCA, bladder urothelial carcinoma; BRCA, breast invasive carcinoma; CESC, cervical squamous cell carcinoma and endocervical adenocarcinoma; COAD, colon adenocarcinoma; ESCA, oesophageal carcinoma; GBM, glioblastoma multiforme; HNSC, head and neck squamous cell carcinoma; KIRC, kidney renal clear cell carcinoma; KIRP, kidney renal papillary cell carcinoma; LAML, acute myeloid leukaemia; LGG, brain lower grade glioma; LIHC, liver hepatocellular carcinoma; LUAD, lung adenocarcinoma; LUSC, lung squamous cell carcinoma; OV, ovarian serous cystadenocarcinoma; PAAD, pancreatic adenocarcinoma; PCPG, pheochromocytoma and paraganglioma; SARC, sarcoma; SKCM, skin cutaneous melanoma; STAD, stomach adenocarcinoma; TGCT, testicular germ cell tumours; THCA, thyroid carcinoma; UCEC, uterine corpus endometrial carcinoma. **(D)** Expression profiles of *STAG2* when it acquires damaging or LoF alterations as compared to when it is WT in cancer cell lines from the Cell Line Project (CLP, http://cancer.sanger.ac.uk/cell_lines) and in TCGA samples and. **(E)** Expression profiles of *STAG1* when *STAG2* acquires damaging or LoF alterations as compared to when it is WT in CLP cell lines and in TCGA samples. The numbers of mutated samples or cell lines are reported in brackets. Distributions were compared using the Wilcoxon test and corresponding p-values are shown; ns = not significant.

*STAG1* and *STAG2* encode two proteins with 70% amino acid identity and the same domain organisation (Figure 1A). These proteins are mutually exclusive subunits of two distinct cohesin complexes – cohesin SA1 and cohesin SA2 – that have undergone partial subfunctionalization while still preserving overlapping functions [3] (Figure 1B). For example, both complexes mediate chromatid cohesion along chromosome arms [11, 12], while cohesin SA1 and cohesin SA2 tether telomeric and centromeric sister chromatids, respectively [11]. Additionally, the two complexes play both overlapping and distinct roles in gene expression regulation [12] and in DNA damage checkpoint in response and repair [13]. Interestingly, the depletion of *STAG1* in HeLa cells results in increased *STAG2* expression and *vice versa*, thus suggesting some degree of compensation between the two genes [13]. However, so far the functional interaction between these two genes has not been investigated in detail.

Both *STAG1* and *STAG2* acquire somatic LoF alterations (homozygous gene deletions, truncating mutations and multiple hits; see Methods) as well as putative damaging missense and splicing mutations in a variety of human cancers of The Cancer Genome Atlas (TCGA, Figure 1C). However *STAG2*, but not *STAG1*, has been identified as a tumour suppressor in leukaemia, sarcoma, glioblastoma and bladder cancer [14–20] (Table 1). This may be due to the localisation of *STAG2* on the X chromosome that makes a single hit sufficient to inactivate the gene. LoF alterations are clearly associated with a significant reduction of *STAG2* expression in cancer cell lines and in TCGA samples (Figure 1D), supporting a reduced gene activity after somatic inactivation. Also, *STAG1* expression slightly increases in cell lines with LoF alterations in *STAG2*, although this is not observed in human samples (Figure 1E). Therefore, despite the effects of *STAG2* depletion on *STAG1* expression seem context-specific (see also below), in some cases it may lead to increased levels of *STAG1*. This, coupled with high sequence identity and partially overlapping functions, suggests a functional compensation, and possibly a synthetic lethal interaction, between the two genes.

**Table 1:** Cancer driver role and paralogy relationship of human cohesin subunits

Cohesin subunit	Cancer type	Reference(s)	Paralog(s)
SMC3	Acute myeloid leukaemia	[7, 58, 59]	No
SMC1A	Urothelial bladder	[14]	SMC1B*
	Acute myeloid leukaemia	[7, 59]	
RAD21	Acute myeloid leukaemia	[59, 60]	RAD21L*
STAG2	Urothelial bladder	[14–17]	STAG1, STAG3*
	Acute myeloid and lymphoblastic leukaemia	[7, 59, 61, 62]	
	Paediatric and adult Ewing sarcoma	[18, 19, 63]	
	Glioblastoma	[20]	

Reported are the cancer types and associated studies where somatic modifications of the cohesin subunit are cancer drivers. * = active in meiosis.

### Transient gene knockdown validates synthetic lethality between *STAG1* and *STAG2*

**Figure 2:**
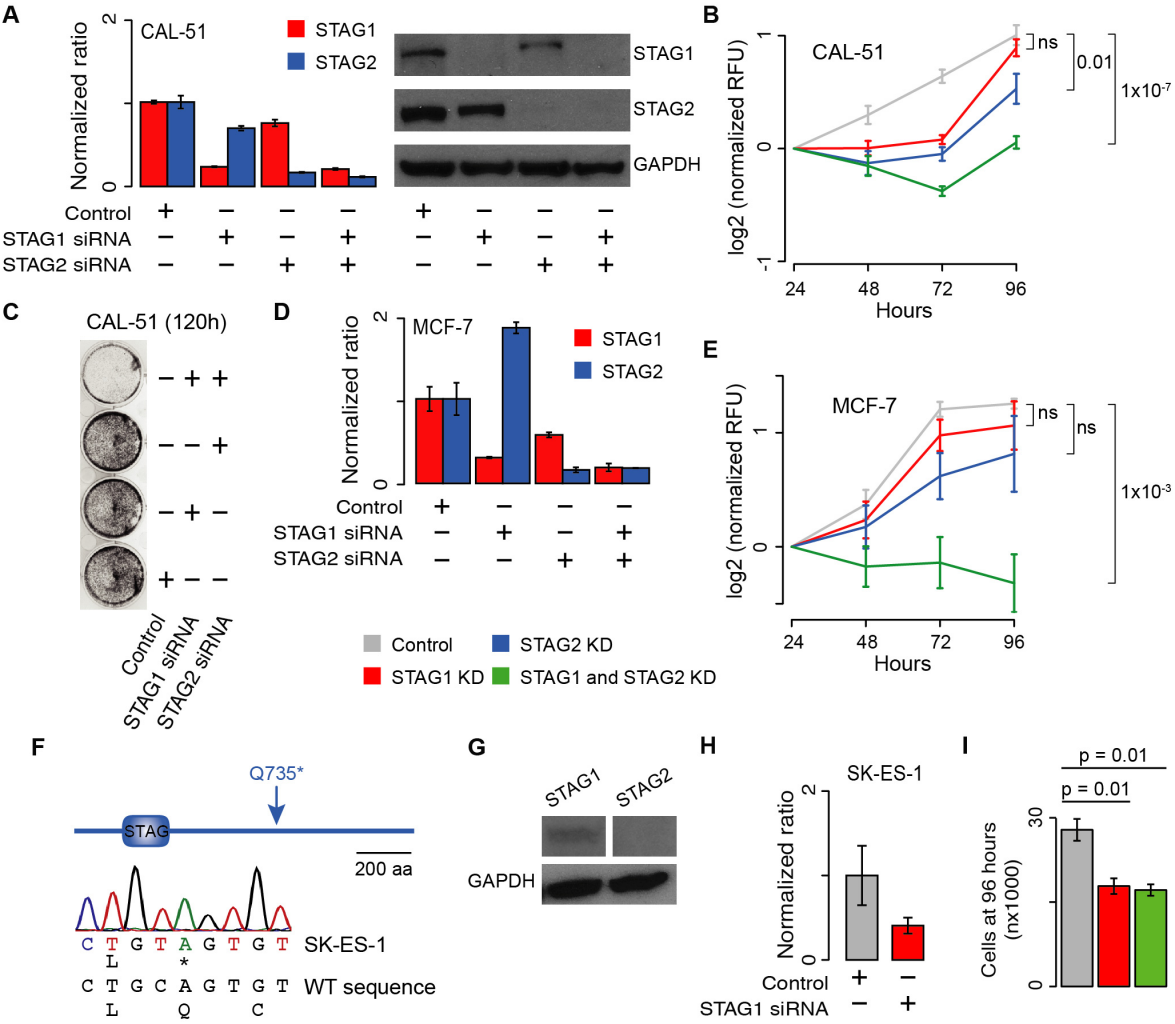
Effect of transient blocking of *STAG1* and *STAG2* on cell proliferation **Legend: (A)** *STAG1* and *STAG2* gene (left) and protein (right) expression in CAL-51 cells 48 hours and 72 hours after siRNA transfection, respectively. **(B)** Proliferation curve of CAL-51 cells after transfection with negative, STAG1 and STAG2 siRNAs. Three biological replicates were done and the KD was repeated three times in each replicate. **(C)** Crystal violet staining of CAL-51 cells 120 hours after transfection with negative, STAG1 and STAG2 siRNAs. **(D)** *STAG1* and *STAG2* expression measured by quantitative RT-PCR in MCF-7 cells 72 hours after siRNA transfection. **(E)** Proliferation curve of MCF-7 cells after transfection with negative, STAG1 and STAG2 siRNAs. Two biological replicates were done and the KD was repeated three times in each replicate. **(F)** Sanger sequencing confirmation of *STAG2* homozygous nonsense mutation in SK-ES-1 cells. **(G)** Immunoblots of STAG1 and STAG2 protein expression in untreated SK-ES-1 cells. **(H)** STAG1 mRNA expression in SK-ES-1 cells after siRNA transfection as compared to the control. **(I)** Number of SK-ES-1 cells 96 hours after transfection of STAG1 or STAG1 and STAG2 siRNAs as compared to the control. Each KD was repeated six times and cells were counted blindly and independently. Average number of cells and associated standard errors across replicates for each condition are shown. Means were compared using one-tailed Student's t-test. In all quantitative RT-PCR experiments, β-2-microglobulin was used for normalisation. Shown are mean and standard error of normalized expression values across replicates. In all proliferation curves, Relative Fluorescent Unit (RFU) values were normalised to the mean across replicates at 24 hours. Mean values at 96 hours were compared using the one-tailed Student's t-test; ns = not significant.

To validate the predicted synthetic lethal interaction between *STAG1* and *STAG2*, we inactivated them in the CAL-51 cancer cell line where both genes are WT (Supplementary Table 1). We knocked down each gene individually or both simultaneously with gene-specific short interfering RNAs (siRNAs), using negative siRNAs as a control (Supplementary Table 2). First, we confirmed decreased mRNA levels and undetectable protein expression of the knocked down genes (Figure 2A). Then we measured cell proliferation 24, 48, 72 and 96 hours after transfection. To ensure that these measures were comparable across replicates and independent of the number of initially seeded cells in each condition, we normalised each time point to the value measured at 24 hours. We found that viable cells after the simultaneous silencing of *STAG1* and *STAG2* were significantly lower than the control (Figure 2B), supporting synthetic lethality between the two genes. Crystal violet staining of CAL-51 cells 120 hours after transfection confirmed that the double knockdown (KD) of *STAG1* and *STAG2* led to a drastic reduction in the number of cells (Figure 2C). To test whether the observed effect was universal or rather specific to CAL-51 cells, we repeated the KD experiment in MCF-7 breast cancer cells, another cell line in which both genes are WT (Supplementary Table 1). Interestingly, *STAG2* expression in MCF-7 cells increased when *STAG1* was knocked down (Figure 2D), in agreement with what has been previously reported in HeLa cells [13]. However, the same signal was not observed in CAL-51 cells (Figure 2A), suggesting that the changes in the relative expression of the two genes are context-specific. We then monitored the effect of the individual or simultaneous blocking of *STAG1* and *STAG2* on MCF-7 cell proliferation as compared to the control. As we found for CAL-51, the proliferation of MCF-7 was significantly impaired in the presence of the double KD of *STAG1* and *STAG2* (Figure 2E). Finally, we tested whether the inhibition of *STAG1* alone was enough to reduce cell proliferation when *STAG2* was already inactive. This condition mimics that of cancer samples where *STAG2* is somatically inactivated and supports the development of *STAG1* as a therapeutic target in these tumours. To test this, we used SK-ES-1, a sarcoma cell line with a somatic homozygous point mutation in *STAG2* (Supplementary Table 1). This mutation introduces a premature stop codon (Figure 2F) resulting in the abolishment of the full-length STAG2 protein expression (Figure 2G). We knocked down *STAG1* via siRNA (Figure 2H) and monitored its effect on cell growth. However, the measure of SK-ES-1 cell proliferation via enzymatic activity yielded inconsistent results even for untreated cells (Supplementary Figure 1A). This is likely because SK-ES-1 cells tend to form aggregates (Supplementary Figure 1B) that prevent uniform incorporation of the reagents needed for the assay. Therefore, instead of measuring cell proliferation, we counted the number of SK-ES-1 cells 96 hours after transfection with negative siRNAs or *STAG1* siRNA. Each transfection was repeated six times and cells were counted blindly and independently by two operators. We found that the number of cells after of *STAG1* KD was significantly lower than the control (Figure 2I). Although we did not detect any full-length protein (Figure 2G), *STAG2* mRNA is still expressed in SK-ES-cells according to the gene expression profile of the cancer cell line project [21]. To rule out the possibility that a truncated version of STAG2 protein was still functional, we knocked down both genes. We confirmed that the simultaneous KD of *STAG1* and *STAG2* had no further detrimental effect on the proliferation of SK-ES-1 cells (Figure 2I). Therefore, the blocking of *STAG1* alone is sufficient to impair cell growth when *STAG2* is inactive.

### Stable gene knockout of *STAG1* and *STAG2* confirms synthetic lethality

**Figure 3:**
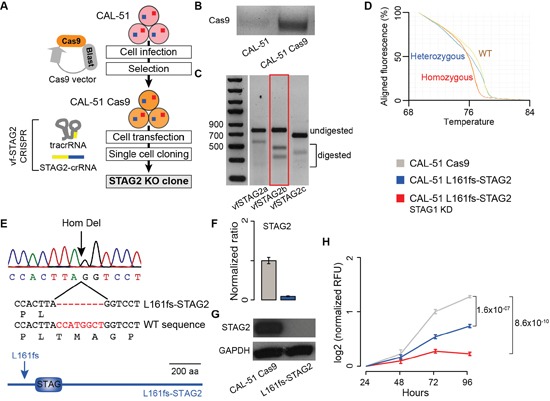
Synthetic lethality between STAG1 and STAG2 in stably edited *STAG2* cells **Legend: (A)** Schematic diagram to derive *STAG2* KO clones via vector-free (vf) CRISPR editing. CAL-51 cells are first infected with a Cas9 containing lentiviral vector to induce Cas9 expression and then transfected with a universal trans-activating RNA (tracrRNA) and gene-specific CRISPR targeting RNAs (crRNAs). Finally, edited clones are isolated via single cell cloning. **(B)** Evidence of Cas9 mRNA expression in CAL-51 Cas9 cells. **(C)** T7 endonuclease 1 assay (T7E1) assay on the edited regions of STAG2 after transfection with three STAG2-crRNAs. STAG2b (red box) was selected because of its higher editing efficiency. **(D)** High Resolution Melting Assay on isolated clones after single cell cloning from a heterogeneous population of *STAG2* edited cells. The assay was used to identify clones with homozygous STAG2 editing. **(E)** Sanger sequencing confirmation of the eight-base-pair-long homozygous deletion in CAL-51 L161fs-STAG2 cells. **(F)** Expression of *STAG2* via quantitative RT-PCR in CAL-51 L161fs-STAG2 cells and in CAL-51 Cas9 cells. β-2-microglobulin was used for normalisation. Shown are mean and standard error of normalized expression values across replicates. **(G)** Western blots of STAG2 protein expression in CAL-51 L161fs-STAG2 cells and CAL-51 Cas9 cells. **(H)** Proliferation curve of CAL-51 L161fs-STAG2 cells after transfection with negative or STAG1 siRNAs. Three biological replicates were done and the KD was repeated three times in each replicate. Relative Fluorescent Unit (RFU) values were normalised to the mean across replicates at 24 hours. Mean values at 96 hours were compared using the one-tailed Student's t-test.

Next, we tested whether the synthetic lethal interaction between *STAG1* and *STAG2* could be confirmed through stable knockout (KO) of *STAG1* or *STAG2* in addition to transient KD of the respective paralog. To stably inactivate *STAG2*, we developed a vector-free (vf) CRISPR system (Figure 3A). First, we induced Cas9 expression in CAL-51 cells (Figure 3B). We then transfected CAL-51 Cas9 expressing cells with a universal trans-activating RNA (tracrRNA) and three different STAG2-specific CRISPR targeting RNAs (crRNAs, Supplementary Table 2). We selected the optimal STAG2 crRNA based on editing efficiency (Figure 3C) and used it in further experiments. Starting from a heterogeneous population of STAG2-edited cells, we performed single cell cloning and identified a clone with homozygous *STAG2* editing using the High Resolution Melting assay (Figure 3D). Sequencing of the edited region confirmed a homozygous eight-nucleotide-long deletion producing a frameshift in the STAG2 protein after Leucine 161 (L161fs-STAG2, Figure 3E). We detected no STAG2 mRNA (Figure 3F) or protein (Figure 3G) expression in CAL-51 L161fs-STAG2 cells. To test the synthetic lethal interaction between *STAG1* and *STAG2* in the presence of stably inactivated *STAG2*, we transfected CAL-51 L161fs-STAG2 cells with either *STAG1* siRNA or negative siRNA. We found that cell proliferation 96 hours after transfection with *STAG1* siRNA was significantly lower than the control (Figure 3H).

**Figure 4:**
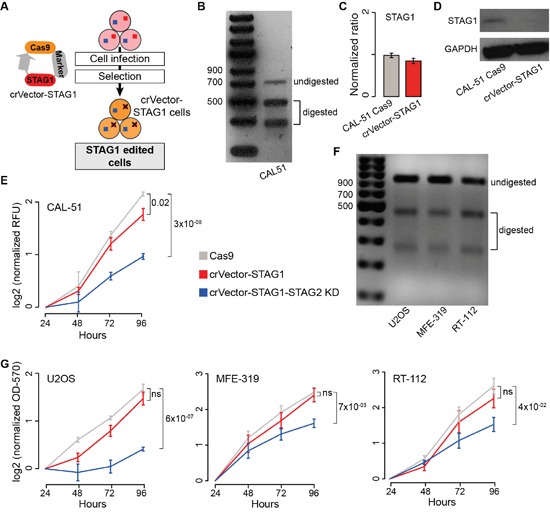
Synthetic lethality between STAG1 and STAG2 in stably edited STAG1 cells **Legend: (A)** Schematic representation of vector-mediated *STAG1* editing. Cells were infected (CAL-51) or transfected (U2OS, MEF-319, RT-112) with a STAG1-Cas9 vector. Resulting STAG1 edited cells were subsequently isolated (see Methods). **(B)** T7E1 assay on STAG1 edited region in crVector-STAG1 CAL-51 cells. **(C)** STAG1 expression in CAL-51 Cas9 and crVector-STAG1 cells. β-2-microglobulin was used for normalisation. Shown are mean and standard error of normalized expression values across replicates. **(D)** Western blots of STAG1 in CAL-51 Cas9 cells and crVector-STAG1 cells. **(E)** Proliferation curve of CAL-51-Cas9 and crVector-STAG1 cells after transfection with negative or STAG2 siRNAs. **(F)** T7E1 assay on STAG1 edited region in crVector-STAG1 U2OS, MFE-319, and RT-112 cells, respectively. **(G)** Proliferation curve of U2OS, MFE-319, and RT-112-Cas9 and corresponding crVector-STAG1 cells after transfection with negative or STAG2 siRNAs. All proliferation assays were done in triplicates, except for MFE-319 where two replicates were performed, and the KD was repeated three times in each replicate. Relative Fluorescent Unit (RFU) values or Optical Density at 570 nm (OD-570) values were normalised to the mean across replicates at 24 hours and log2 transformed. Mean values at 96 hours were compared using the one-tailed Student's t-test; ns = not significant.

To stably inactivate *STAG1*, we infected CAL-51 cells with a lentiviral vector containing Cas9*sp*, an antibiotic resistance marker, and a STAG1 guide RNA (gRNA, Figure 4A, Supplementary Table 2). After antibiotic selection, we obtained *STAG1* CAL-51 edited cells (CAL-51 crVector-STAG1, Figure 4B). Although STAG1 mRNA level in CAL-51 crVector-STAG1 cells was comparable to the control (Figure 4C), no STAG1 protein was detected (Figure 4D). We then transfected CAL-51 crVector-STAG1 cells directly with either *STAG2* siRNA or negative siRNA. Again, we found that the silencing of *STAG2* significantly reduced the proliferation of crVector-STAG1 cells as compared to the control (Figure 4E). Using a similar approach, we generated stable *STAG1* KO in sarcoma (U2OS), uterine carcinoma (MFE-319) and bladder carcinoma (RT-112) cell lines that have both genes WT, but with a different number of copies (Supplementary Table 1). We first induced STAG1 editing (Figure 4F) and then transfected the cells with *STAG2* or control siRNAs, observing significant reduction of cell proliferation only when both genes were blocked (Figure 4G).

**Figure 5:**
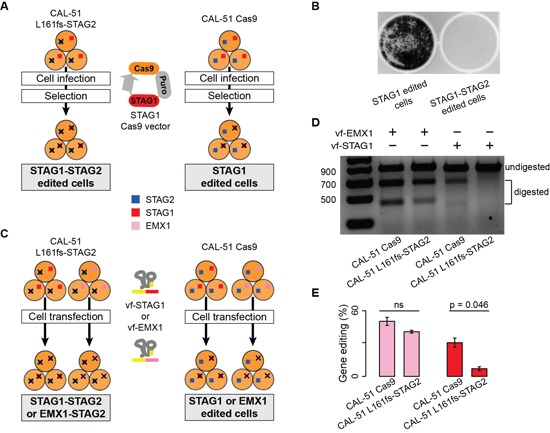
Synthetic lethality between STAG1 and STAG2 via double gene editing **Legend: (A)** Schematic representation of *STAG1* and *STAG2* double gene editing using a lentiviral vector. CAL-51 L161fs-STAG2 and CAL-51 Cas9 cellswere infected with the STAG1-Cas9 lentiviral vector and subjected to puromycin selection to produce *STAG1-STAG2* and *STAG1* edited cells, respectively. **(B)** Crystal violet staining of CAL-51 *STAG1* and *STAG1*-*STAG2* edited cells ten days after puromycin selection. Less than 150 cells were counted in CAL-51 *STAG1*-*STAG2* edited cells as compared to around 200,000 CAL-51 *STAG1* edited cells. **(C)** Schematic representation of the *STAG1* and *STAG2* double gene editing using the vf-CRISPR system. *STAG1* or *EMX1* were edited using the vf-CRISPR system on CAL-51 L161fs-STAG2 and CAL-51 Cas9 cells to generate STAG1-STAG2 or EMX1-STAG2 or STAG1 or EMX1 edited cells, respectively. **(D)** Representative T7E1 assay on *EMX1* and *STAG1* edited regions in CAL-51 Cas9 and CAL-51 L161fs-STAG2 cells. **(E)** Quantification of *EMX1* and *STAG1* gene editing in CAL-51 Cas9 and CAL-51 L161fs-STAG2 cells. Each gene editing was repeated three times and each time the percentage of editing was quantified using ImageJ. Barplots show the mean percentage of gene editing and associated standard errors across replicates. One-tailed Student's t-test was used to assess statistical significance and corresponding p-values are shown; ns = not significant.

As a final validation, we measured the effect of the stable KO of both *STAG1* and *STAG2* in two different experimental settings. In the first experimental setting, we compared the growth of CAL-51 L161fs-STAG2 cells and CAL-51 Cas9 cells after infection with the STAG1 Cas9 lentiviral vector (Figure 5A). Crystal violet staining showed that while STAG1 edited cells reached around 75% confluence after ten days of antibiotic selection, the double editing of both genes critically reduced the number of cells (Figure 5B). In the second experimental setting, we used the vf-CRISPR system to edit *STAG1* in both CAL-51 L161fs-STAG2 cells and CAL-51 Cas9 cells (Figure 5C). Here the expectation was that because of synthetic lethality, *STAG1* editing should be significantly less efficient in CAL-51 L161fs-STAG2 cells as compared to CAL-51 Cas9 cells. As we did for STAG2, we tested three different STAG1 crRNAs and used the one with the highest editing efficiency (Supplementary Figure 2A). Moreover, we added Cas9 protein to the transfection mix after verifying that this further improves the editing efficiency (Supplementary Figure 2B). As a further control to rule out the possibility that *STAG2* KO would interfere with any additional gene editing, we edited *EMX1*, an unrelated gene that is broadly used as a positive control for CRISPR-induced gene editing [22, 23], in both cell lines under the same conditions used to edit *STAG1* (Figure 5C). While we observed comparable *EMX1* editing efficiency in CAL-51 L161fs-STAG2 cells and in CAL-51 Cas9 cells, *STAG1* editing was clearly detectable in CAL-51 Cas9 cells but almost absent in CAL-51 L161fs-STAG2 cells (Figure 5D). Quantification of the gel bands corresponding to the edited regions confirmed no difference in the fraction of *EMX1* editing and a significantly lower fraction of *STAG1* editing in CAL-51 L161fs-STAG2 cells as compared to CAL-51 Cas9 cells (Figure 5E). This excludes the possibility that CAL-51 L161fs-STAG2 cells are resistant to additional gene editing and proves specific counter selection of *STAG1* and *STAG2* simultaneous KO due to the synthetic lethality between the two genes.

## DISCUSSION

The cohesin complex has recently attracted increased attention as an interesting therapeutic target because of its frequent somatic inactivation in cancer. For example, it has been shown that, STAG2-deficient glioblastoma cells, although not STAG2-deficient Ewing sarcoma cells [24], are more responsive than STAG2 proficient cells to treatment with PARP inhibitors [25]. Synthetic lethality between cohesin and PARP has been explained by their respective roles in recovering and maintaining the integrity of stalled replication forks [26]. In the presence of defective cohesin, cancer cells become dependent on replication fork mediators, such as PARP, to replicate the genome correctly and efficiently [27]. When these mediators are also inhibited, the replication fork cannot progress resulting in double strand breaks [26]. This is an example of synthetic lethality resulting from the concomitant inhibition of two independent pathways that are nonessential *per se* but that both contribute to an essential process – in this case DNA replication. Cells with altered cohesin are also sensitive to the inhibition of the anaphase promoting complex/cyclosome (APC/C), which is required for exiting mitosis after proper chromosome segregation [28]. In this case, synthetic lethality between cohesin and APC/C derives from the additive effect of their simultaneous inhibition, namely weak chromatid cohesion and delayed exit from mitosis [28].

Here we describe yet another mechanism of synthetic lethality involving cohesin that results from the ability of two paralogous genes, *STAG1* and *STAG2*, to compensate for each other. This is due to the gene common evolutionary origin, high sequence conservation and partial retention of original functions. In several cancer types, the somatic inactivation of *STAG2* is selected for due to its tumour suppressor role. However, this yields mutated cells that become dependent on *STAG1* and sensitive to its inhibition. Our data on SK-ES-1 cells, which have inactive *STAG2* gene, show that this vulnerability can be exploited to specifically target STAG2-altered cancers by developing specific STAG1 inhibitors. Moreover, it has been recently reported that *STAG2* inactivation confers resistance to BRAF inhibitors in melanoma [29]. The vulnerability of *STAG2*-deficient cells towards STAG1 inhibition may help overcome the onset of this resistance. Paralog dependencies that create cancer vulnerabilities have already been described for another chromatin-related complex, the SWI/SNF complex. In this case, cancer cells lacking *SMARCA4* or *ARID1A*, two core components of the SWI/SNF complex, become dependent on the corresponding paralogs, *SMARCA2* and *ARID1B*, respectively [30–33]. As a result, the SWI/SNF complex has attracted a great deal of attention for the development of targeted cancer therapies [34, 35]. Our results suggest that paralog dependency is a general mechanism to buffer single gene loss. As such, it is a powerful strategy to discover cancer vulnerabilities [10].

Synthetic dependencies are often difficult to validate experimentally because of their context specificity, partial silencing and widespread off-target effects of common approaches based on RNA interference [9]. To prove that the synthetic lethality between *STAG1* and *STAG2* is context independent, we have tested their genetic interaction in several cancer cell lines where the two genes have variable mutational and copy number status. As already reported in the literature [14, 16, 36], we confirm that the impairment of either STAG paralog alone has a context-specific effect on cell proliferation. In our experiments, blocking *STAG1* or *STAG2* has either no effect or can slightly reduce cell proliferation. However, the concomitant blockade of both genes leads to substantially lower cell growth in all cell lines that we have tested. Moreover, we have induced gene inhibition with both RNA interference and gene editing, obtaining comparable results in all cases. Our vf-CRISPR-based system induces stable gene KO through the formation of a transient Cas9-crRNA-tracrRNA complex. This represents a major advantage to reduce off-target effects that are likely to occur when all CRISPR-Cas9 components are stably expressed [37]. Similar vf-CRISPR approaches have recently been used to induce gene editing *in vitro* [38–40] and *in vivo* [41–43]. In most of these studies, gRNAs are first generated via *in vitro* transcription and then transfected with Cas9 into the cells. Alternatively, all CRISPR-Cas9 components are injected directly into the cells. Here, we further simplify this approach to prove that a simple transfection is able to induce editing of single and multiple genes and can be efficiently applied to prove genetic interactions.

## MATERIALS AND METHODS

### *STAG1* and *STAG2* somatic alterations in TCGA and cancer cell line project

Somatic mutations (single nucleotide variants and small indels), segmented copy numbers and RNA sequencing data were downloaded from TCGA Data Matrix portal (Level 3, https://tcga-data.nci.nih.gov/docs/publications/tcga/) for 31 cancer types. Only non-hypermutated samples with a number of mutations within the third quartile of the distribution of mutations for the corresponding cancer type were further retained. *STAG1* and *STAG2* were considered as somatically inactive if they acquired LoF alterations or damaging alterations. LoF alterations were identified as homozygous gene deletions, truncating mutations (stopgain, stoploss, frameshift indels) or multiple hits (combination of heterozygous gene deletions, truncating and damaging mutations). Damaging alterations were defined as missense and splicing mutations with predicted damaging effects on the protein. Missense mutations were considered damaging if supported by at least five out of eight function-based scores (SIFT [44], PolyPhen-2 HDIV [45], PolyPhen-2 HVAR [45], MutationTaster [46], MutationAssessor [47], LTR [48] and FATHMM [49]) or two out of three conservation-based scores (PhyloP [50], GERP++ RS [51], SiPhy [52]). Splicing mutations were predicted as damaging if supported by at least one ensemble score of dbscSNV [53]. *STAG1* and *STAG2* were considered deleted if their copy numbers (CN) were <0.5 (homozygous deletion) or <1.5 (heterozygous deletion). CN was measured as

$$CN=2^{segment\;mean}x2$$

where segment mean is the segment copy number of STAG1 or STAG2 genomic regions.

To compare the expression of mutated and WT *STAG1* and *STAG2*, we converted either the Expectation-Maximization (RSEM) or the Reads Per Kilobase per Million mapped reads (RPKM) into transcripts per million (TPM). Starting from RPKM, TPM were calculated as:

$$TPM\left(STAG1,\;STAG2\right)=\frac{RPKM\left(STAG1,\;STAG2\right)}{\sum_{i}RPKM\left(STAG1,\;STAG2\right)}\;x\;10^{6}$$

RSEM were multiplied by one million to obtain the corresponding TPM.

STAG1 and STAG2 somatic mutations and Affymetrix U219 expression data in 971 cancer cell lines were obtained from the cancer Cell Lines Project (CLP, http://cancer.sanger.ac.uk/cell_lines). LoF alterations were defined as stopgain, stoploss and frameshift indels. Damaging mutations were identified from the CLP annotation. The expression levels of STAG1 and STAG2 were derived from CLP and measured as Z-scores of Robust Multiarray Average (RMA)-normalised expression values [54].

### Cell lines

The cell lines used in this study (CAL-51, MCF-7, SK-ES-1, U2OS, MFE-319 and RT-112) were all validated by short tandem repeat analysis. Cells were grown at 37°C and five per cent CO_2_ in DMEM 10% FBS (CAL-51, U2OS), DMEM 10% FBS 0.01 mg/ml human recombinant insulin (MCF-7), McCoy's 20% calf serum (SK-ES-1), DMEM-RPMI 20% FBS (MFE-319) and RPMI 10% FBS (RT-112). To confirm the *STAG2* mutation in SK-ES-1 cells, genomic DNA was extracted using GenElute mammalian genomic DNA miniprep kit (Sigma-Aldrich) according to the manufacturer's protocol. Sanger sequencing was performed after PCR amplification of a 634-base-pair-long genomic region surrounding the *STAG2* mutated position as annotated in CLP (Supplementary Table 2).

### siRNA transfection

Transfection was performed with lipofectamine RNAiMAX (Thermo Fisher Scientific) and mission pre-designed siRNA oligos specific for *STAG1* and *STAG2* (Supplementary Table 2) using two universal negative siRNA oligos as controls (Sigma-Aldrich) following the manufacturer's protocol. For proliferation assays, transfections were performed in 96-well plates, while for RNA and protein extraction, transfections were performed in 24-well plates and 6-well plates, respectively. In all assays, the total concentration of the RNAi oligos was 50 nM (25 nM for each siRNA). For the double KD, the two *STAG1* and *STAG2* siRNA oligos were mixed. For the single KD, the siRNA specific either for STAG1 or STAG2 was combined with one of the universal negative siRNA oligos. For the control, the two universal negative siRNA oligos were used.

### Quantitative RT-PCR

Total RNA was extracted using GenElute mammalian total RNA miniprep kit (Sigma-Aldrich). Reverse transcription was performed starting from 175 ng RNA using GoScript^TM^ reverse transcription system (Promega). cDNAs were subjected to quantitative PCR using predesigned SYBR green primers (Sigma-Aldrich; Supplementary Table 2) and SYBR Green JumpStart Taq ReadyMix^TM^ (Sigma-Aldrich). Gene expression levels were assessed in triplicate using ViiA7 thermal cycler (Applied Biosystems) and the average expression level across triplicates (*e*) was relativized to the average expression level of β-2-microglobulin (*c*):

r = e − c

where *r* is the relative gene expression.

The fold change (*fc*) between the relative gene expression after KD (*r_KD_*) and the relative gene expression in the control condition (*r*_c_) was calculated as:

$$f_{c}\;=\;{\text{2}}^{\left(r_{c}\;-\;rKD\right)}$$

Each experiment was repeated in biological duplicate.

### Western blot analysis

CAL-51 cells were seeded at 70% confluence in 60 mm plates, grown for 48 hours after gene KD or for 24 hours after CRISPR editing, washed twice with PBS and lysed with RIPA buffer. Protein amounts present in the cell lysates were measured using Pierce BCA protein assay kit (Thermo Fisher Scientific). Five to ten micrograms of protein were loaded in TruPAGE^TM^ precast gels (Sigma-Aldrich). STAG1, STAG2 or GAPDH proteins were detected by incubating membranes overnight with anti-STAG1 prestige rabbit antibody at 1/150 dilution (Sigma Life Science, HPA035015), anti-STAG2 mouse antibody 1/500 dilution (Sigma-Aldrich, WHO0010735M1) or anti-GAPDH mouse antibody 1/10000 dilution (MAB374, clone 6C5, EMD Millipore), respectively. After washing with 0.01% PBS-Tween 20, membranes were incubated with peroxidase-conjugated anti-mouse or anti-rabbit antibodies (1:5000, Mouse IgG HRP-linked Whole Ab NA934 and Rabbit IgG HRP-linked Whole Ab NA934, GE Healthcare) for 45 minutes and washed before detection by chemiluminescence (ECL, GE Healthcare).

### Proliferation assays and crystal violet staining

Cell proliferation was measured every 24 hours for four days, starting one day after transfection using either CellTiter-FluorTM cell viability assay (Promega) or crystal violet staining followed by dye extraction using methanol and optical density measured at 570 nm [55]. Briefly, 5×10^3^ cells/well transfected with STAG1, STAG2 or negative siRNAs were seeded on 96-well plates in a final volume of 100 μl per well. For the CellTiter-FluorTM cell viability assay (Promega), at each time point, 20 μl of the diluted reagent (10 μl of the GF-AFC Substrate in 2 ml of Assay Buffer) was added to each well. After one hour and 30 minutes, fluorescence was measured at 380–400 nm_Ex_/505_Em_ using a Fusion alpha-FP (Perkin Elmer). Each condition was assessed in triplicate and the whole experiment was repeated at least twice. Crystal violet staining was used to visualise the effect of *STAG1* and *STAG2* KD on CAL-51 cells. Briefly, 70000 cells were seeded on 12-well plates and transfected with the universal negative control, STAG1, STAG2 or STAG1 and STAG2 siRNAs. After five days, cells were fixed with ice-cold 100% methanol and stained with 0.1% crystal violet. After 30 minutes, cells were washed three times with water and dried.

### *STAG1* editing with CRISPR vectors and T7E1 assay

CAL-51 cells were transduced with the lentiviral vector (pLV-U6g-EPCG) containing Cas9sp, the puromycin resistance marker and a STAG1 gRNA (Sigma-Aldrich). The gRNA was composed of a universal 86-nucleotide-long tracRNA and 20-nucleotide-long STAG1-specific crRNA (Supplementary Table 2). Forty-eight hours after infection, stably transduced cells were selected with puromycin (6μg/ml). U2OS, MFE-319 and RT-112 cells were transfected with a vector containing Cas9sp, the Orange Fluorescent Protein (OFP) reporter (GeneArt Nuclease, Thermo Fisher Scientific) and the STAG1 gRNA1a (Supplementary Table 2). Forward and reverse strand oligos corresponding to STAG1 gRNA1a were synthesized with 3’overhangs nucleotides according to the manufacturer's protocol. Resulting oligos were annealed and cloned into the linearized GeneArt Nuclease vector. After verification of STAG1 gRNA integration via Sanger sequencing, cells were transfected using Fugene6 (Promega) and OFP positive cells were sorted after 72 hours. The per cent of edited cells was assessed two weeks after selection or one week after OFP positive cell sorting using the T7 endonuclease 1 (T7E1) assay (New England Biolabs), following the manufacturer's protocol. Briefly, the genomic site targeted by the STAG1 crRNAs was amplified from 100 ng of genomic DNA extracted with GenElute genomic DNA extraction kit (Sigma-Aldrich), using Q5 Hot Start High-Fidelity 2X Master Mix (New England Biolabs) and specific primers (Supplementary Table 2). Amplicons of 700-800 base pairs were purified using GenElute PCR clean-up kit (Sigma-Aldrich) and 200 ng of PCR products were denatured, annealed and digested with the T7 endonuclease 1 enzyme (New England Biolabs) for 15 minutes. Digested products were run on a two per cent agarose gel and the intensity of bands corresponding to the full-length amplicon and, in presence of editing, the two digested fragments were quantified with ImageJ [56] and GelAnalyzer (http://www.gelanalyzer.com/index.html). Each band was quantified three times and the whole experiment was repeated three times.

### Generation of CAL-51-Cas9 expressing cells and gene editing with vector-free CRISPR

CAL-51 cells were transduced with a lentiviral vector containing Cas9*sp* (GeCKO-tEf1aCas9Blast, Sigma-Aldrich) and blasticidin resistance marker. After 10 days of treatment with blasticidin (25 ug/ml), resistant cells were selected and Cas9*sp* expression was verified via PCR (Supplementary Table 2). STAG1 and STAG2 crRNAs (Sigma-Aldrich, Supplementary Table 2) were co-transfected with 69-mer tracrRNA (Sigma-Aldrich) together with GeneArt Platinum Cas9 nuclease (Life Technologies) using lipofectamine CRISPRMAX (Life Technologies). The per cent of edited cells was assessed 72 hours after transfection using the T7E1 assay (New England Biolabs) as reported above.

### Isolation and identification of *STAG2* edited clones

*STAG2* was edited on 200,000 CAL-51 Cas9 expressing cells (CAL-51-Cas9) using the vector-free (vf) CRISPR method and the STAG2 2b crRNA (Supplementary Table 2). Seventy-two hours after editing, cells were dissociated with trypsin, counted and seeded as single cells on 96-well plates. Seven clones were further assessed to evaluate the editing status using High Resolution Melting Assay (HRMA). Genomic DNA was extracted from each clone and from CAL-51-Cas9 cells and a 125-base-pair-long segment surrounding the STAG2b edited region was amplified with specific primers (Supplementary Table 2) using MeltDoctor HRM master mix (Applied Biosystems) following the manufacturer's cycling conditions. The Applied Biosystems High Resolution Melting Software was used to review the melt curves and distinguish between homozygous and heterozygous edited clones as compared to WT. The homozygous deletion of an eight-base-pair-long segment was confirmed with Sanger sequencing (Supplementary Table 2).

## SUPPLEMENTARY MATERIALS FIGURES AND TABLES


