# Supplementary material for: Synthetic lethal interaction between the tumour suppressor STAG2 and its paralog STAG1
Source: Oncotarget. 2017 Apr 5;8(23):37619–32. doi: 10.18632/oncotarget.16838 (PMC5514935; doi:10.18632/oncotarget.16838)
Supplement: Supplementary file 1 [file oncotarget-08-37619-s001.pdf]

Synthetic lethal interaction between the tumour suppressor *STAG2* and its paralog *STAG1*

SUPPLEMENTARY MATERIALS

SUPPLEMENTARY FIGURES AND TABLES

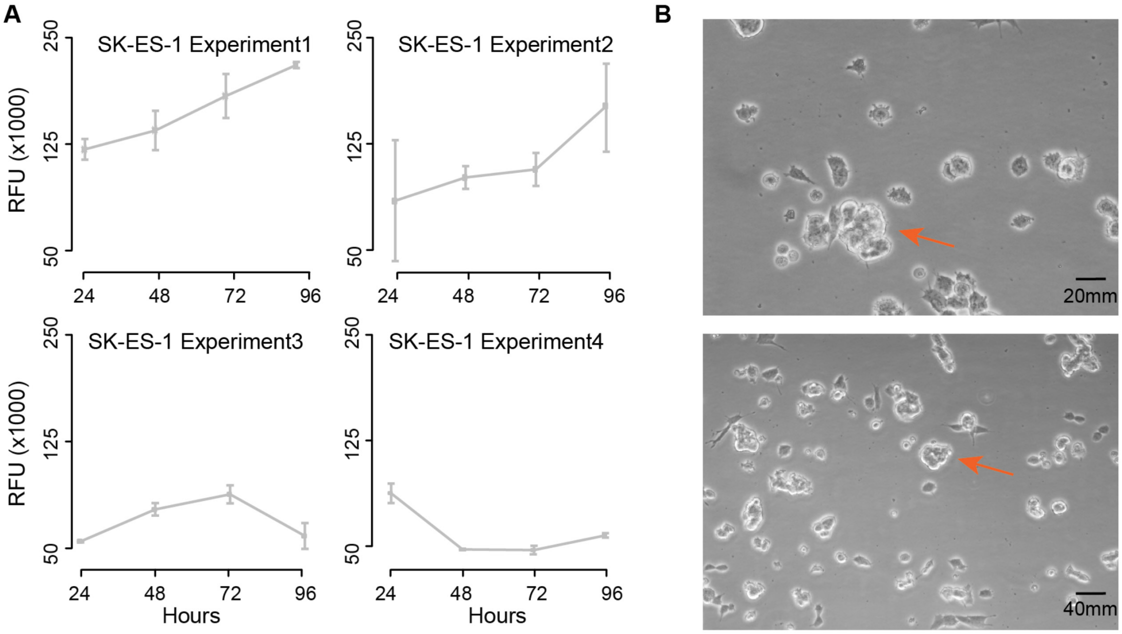

Supplementary Figure 1: SK-ES-1 proliferation curves. Legend: (A) Shown are four proliferation curves of untreated SK-ES-1 cells using CellTiter-Fluor™ cell viability assay (Promega). Each of the four experiments was performed in triplicate starting from 5000 cells. Reported are the average and standard error of the triplicates at each time point. Similar inconsistent results were obtained using Cell counting kit 8 (Sigma-Aldrich, data not shown). **(B)** Representative microscopic images showing SK-ES-1 morphology in cell culture. Red arrows point to cell aggregates.

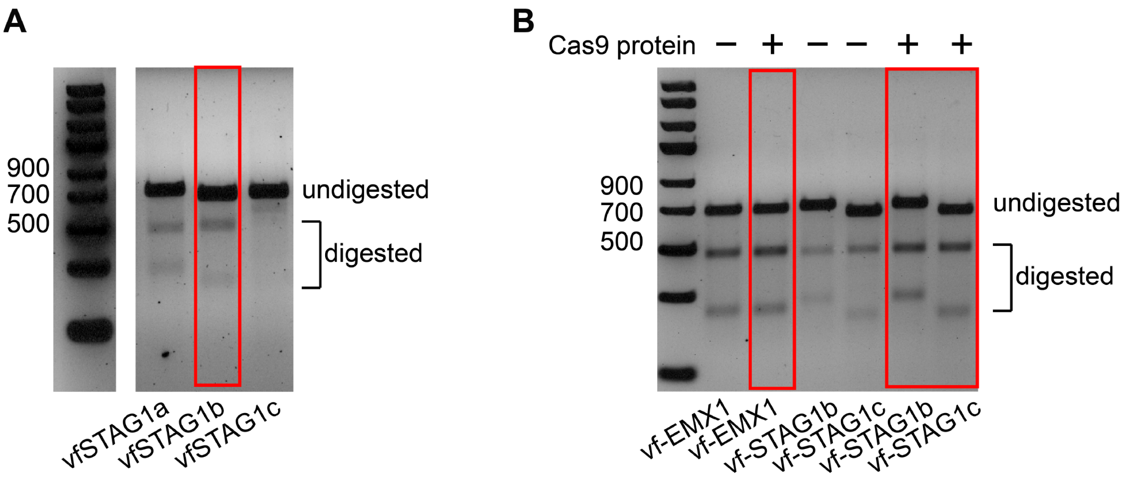

Supplementary Figure 2: Evaluation of STAG1-crRNAs and of Cas9 protein on editing efficiency. Legend: (A) T7E1 assay showing the edited regions of STAG1 after transfection with three different STAG1-crRNAs in CAL-51 Cas9 cells. **(B)** T7E1 assay on the edited regions of STAG1 after transfection with two crRNAs in CAL-51 Cas9 cells with or without the addition of Cas9 protein to the transfection mix. Red boxes highlight the condition leading to the highest editing efficiency.

Supplementary Table 1: *STAG1* and *STAG2* mutations and copy number status in CAL-51, MCF-7, SK-ES-1, U2OS, MFE-319 and RT-112 cells

Cell line	STAG1		STAG2	
	Mutation	Copy number	Mutation	Copy number
CAL-51	WT	2	WT	2
MCF-7	WT	4	WT	3
SK-ES-1	WT	2	Q735*	1
U2OS	WT	3.5	WT	1.44
MFE-319	WT	3	WT	1
RT-112	WT	2.37	WT	2

Legend: Mutations and copy number status in each cell line were derived from the Cell Lines Project (http://cancer.sanger.ac.uk/cell_lines).

Supplementary Table 2: Oligos and primers used in the study

Experiment	Gene	Oligo name	Sequence	Amplicon size
siRNA transfection	STAG1	SASI_Hs02_00340420	GAAAUUGGAGUAUGGAUGA	NA
	STAG1	SASI_Hs02_00340420_As	UCAUCCAUACUCCAAUUU	NA
	STAG2	SASI_Hs02_00311139	GUUCUCACUGCAGAAGAUU	NA
	STAG2	SASI_Hs02_00311139_As	AAUCUUCUGCAGUGAGAAC	NA
Quantitative RT-PCR	STAG1	forward	AGATGAGAGTCTGGATAACAC	157
		reverse	AGAACCATGTTCAGACTCAG	
	STAG2	forward	ATTTGCCACCATCAAAGAAC	79
		reverse	TCATCCATAATTGAAGCTGG	
	Beta-2-microglobulin	forward	AAGGACTGGTCTTTCTATCTC	118
		reverse	GATCCCACTTAACTATCTTGG	
Sanger sequencing in SK-ES-1	STAG2	forward	TACAGTGCCCATGACCTTTC	498
		reverse	CCACAGATTATGCCACCTTCA	
CRISPR Gene editing	EMX-1	NA	GAGTCCGAGCAGAAGAAGAA	NA
	STAG1*	1a	CTACTGCCCATTCCGATGCTGG	NA
	STAG1	1b	CCATTCCGATGCTGGCAGCGAG	NA
	STAG1	1c	AGAAACCTCGAAAATCTCCAGG	NA
	STAG2	2a	CCTTCTGGTCCAAACCGAATGA	NA
	STAG2	2b	GGAGATTATCCACTTACCATGG	NA
	STAG2	2c	ATTCATTGGCGTGTTAGTACGG	NA
T7E1 assay	EMX	forward	ACTCTGTGAAGAAGCGATTATGA	731
		reverse	CTTGTCCCTCTGTCAATGGC	
	STAG1	forward (1a)	AGCTCTTCTAAACTTCCCAATCA	753
		reverse (1a)	AGCAGCTATCATTTCCACAATCA	
	STAG1	forward (1b)	GAGCTCTTCTAAACTTCCCAATC	701
		reverse (1b)	GGGGATTCATTTCACTCACTGT	
	STAG1	forward (1c)	TTTCCCCAGTTTCACCACCT	709
		reverse (1c)	ATGTCAAGAAGGGGCAAGGT	
	STAG2	forward (2a)	CACCACAAAGAGGCTGTCAC	817
		reverse (2a)	ACCCCGTCTCCACTGAAAAT	
	STAG2	forward (2b)	TGACTGTGTGATGTATTTGAAGC	830
		reverse (2b)	CCCATTTCCAGGAATTCGCA	
	STAG2	forward (2c)	TGGTTTGGTTTGGGTTTGCT	710
		reverse (2c)	CCCATTTCCAGGAATTCGCA	
PCR to check Cas9 expression	Cas9	forward	GGGGGACAGTCTTCACGAGC	NA
		reverse	CACGTACATGTCCCTGCCGT	NA
HRMA	STAG2	forward (2b)	CCTTTTCATGCTTTTGTCTAGGG	125
		reverse (2b)	TGACATTGCCGTACTAACACt	

Legend: Reported are the sequences of the DNA and RNA oligos used in this study. NA=not applicable.
